# Effects of Intrahepatic Cholestasis on the Foetus During Pregnancy

**DOI:** 10.7759/cureus.30657

**Published:** 2022-10-25

**Authors:** Anushree Sahni, Sangita D Jogdand

**Affiliations:** 1 Pharmacology, Jawaharlal Nehru Medical College, Datta Meghe Institute of Medical Sciences, Wardha, IND

**Keywords:** total bile acids (tba), ursodeoxycholic acid (udca), intrauterine foetal death (iufd), foetal complications, intrahepatic cholestasis of pregnancy (icp)

## Abstract

The most typical condition of the liver in pregnancy is intrahepatic cholestasis of pregnancy (ICP). There is the occurrence of itching/pruritus together with a decline in liver function tests (LFTs) and frequently higher blood levels of total bile acids, which are used to make the diagnosis. ICP often shows symptoms during the third term of pregnancy and sometimes in the second term. After delivery, the disease's symptoms disappear on their own. It is still unclear what causes this disorder. It constitutes a hazard for the infant and is exceedingly stressful for the mother. Although relatively harmless for the expectant mother, ICP poses a significant risk to the unborn child. Preterm birth, meconium excreted in the amniotic fluid, respiratory distress syndrome, foetal distress and abrupt intrauterine foetal death are all risks seen in this disorder. It is still challenging to identify foetuses who are at risk for ICP issues. There needs to be a clear consensus on the best obstetrical care for ICPs. This review is done to brief the research on the foetal consequences of ICP and to discuss treatment strategies for its avoidance. Serum alanine transaminase, aspartate transaminase, total bilirubin, alkaline phosphatase, albumin, direct bilirubin, total protein, and total bile acids were among the biochemical predictors. Blood tests that confirm obstetric cholestasis should alter the course of treatment. Ursodeoxycholic acid may be prescribed to affected individuals to cure itching and prevent the build-up of biliary components of maternal origin in the baby, which may increase the danger of foetal discomfort and stillbirth.

## Introduction and background

A common disorder of the liver found in pregnancy is intrahepatic cholestasis of pregnancy (ICP) or obstetric cholestasis. It is a cholestatic disease that is reversible and manifests in the second-third term of pregnancy. It is characterized by itching, primarily of the soles and palms, elevated serum bile acid, and elevated transaminases, with spontaneous relief of laboratory anomalies and symptoms that occur as soon as the baby is delivered but no later than 30-days postpartum [1]. It is associated with cholesterol gallstones. It constitutes hazards for the infant and is exceedingly stressful for the mother [2]. According to clinical research, obstetric cholestasis complicating pregnancies can cause up to 60% of preterm deliveries, up to 33% of foetal distress, and up to 2% of infants to die in the womb. Acute anoxia is the main factor in foetal death [3].

## Review

Aetiology

Some of the significant causes of ICP include environmental, hereditary and hormonal variables. Previous research has revealed that the estradiol metabolites, particularly 17-oestradiol glucuronide, have been linked to ICP cholestatic effects. However, progesterone metabolites are considerably more crucial to its pathophysiology. The ratio of 3 α to 3 β hydroxysteroids is much higher in patients suffering from ICP, and in urine, they also excrete a lot of mono- or disulphated progesterone metabolites [4]. An increased percentage of these metabolites in the urine in ICP might be caused by biliary canalicular transporters not functioning correctly, which would typically be in charge of secreting these bioactive components from hepatocytes into bile. Recent research has revealed that people with uncommon, inborn cholestatic disorders like progressive familial intrahepatic cholestasis type 3, which are linked to biliary transporter failure, are more likely to experience ICP. This suggests that mothers heterozygous for mutations in genes coding for transporter proteins are more prone to ICP. For instance, women who were heterozygous for multidrug resistance protein 3 mutation got ICP and had a family history of progressive familial intrahepatic cholestasis type 3 [5]. A mutation linked to the malfunction of bile canalicular phospholipid transporter multidrug resistance protein 3, which leads to elevated amounts of gamma-glutamyl transferase (GGT), was the basis for the infant's disorder. 

Pathophysiology 

The main contributing factors to the development of ICP are genetic predisposition and reproductive hormones, mainly estrogen and progesterone. In genetically susceptible women, estrogen decreases the expression of nuclear hepatic bile acid receptors and hepatic biliary canalicular transport proteins, impairing hepatic bile acid homeostasis and leading to an increase in bile acid levels [6]. A further indication of involvement in pathogenesis is the downregulation of the bile acid transporters organic anion transporting polypeptides human organic anion transporting polypeptide 1A2 (OATP1A2) and human organic anion transporting polypeptide 1B3 (OATP1B3) in ICP placentas [7]. The transport proteins are affected by genetic mutations in the gene coding for transport proteins, steroid hormones, and medications (methyldopa, cetirizine, etc) and accumulations of toxic substances, which negatively affect the expression and function of transport proteins.

The pathogenesis is not clear, and pathogenic alterations to the placenta are inadequate to explain the clinical condition. According to recent research, the placental structural and function damage is due to high bile acid levels, which may be the main cause of perinatal deaths. Foetal development and intrauterine hypoxia can also be influenced by changes in placental structure and function and endocrine alterations. The hazardous effects of bile acid on developing foetuses' hearts, lungs, brains, and other vital organs and hemodynamic changes have been the subject of relevant studies in recent years [8]. As the level of maternal bile acids increases, the risk of foetal complications also increases. Through the placenta, maternal bile acids are transferred to the foetus and deposited in the amniotic fluid to cause abnormalities. Preterm labour is more common in women with ICP; the explanation is unknown, although it might be because of bile acid deposition in the uterine myometrium, which leads to an increase in uterine activity. The most concerning side effect of ICP is sudden intrauterine death. Although the exact cause of foetal death is not known, it may be related to the toxic effects of bile acids on the developing foetus's heart, which can lead to arrhythmias [9], and chorionic vasospasm, which prevents oxygen-rich maternal blood from reaching the developing foetus, which can result in asphyxia.

Clinical findings

Maternal Findings

Pruritus or itching, which often appears in the third term, is the main sign of ICP that occurs most frequently. This worsens during the pregnancy and usually goes away 48 hours after childbirth. An uncomfortable feeling that makes the patient scratch is referred to as pruritus. Although it can affect other body parts, it usually affects the soles and palms. Except for scratch marks, which might be severe, there are no additional dermatological characteristics. Many patients claim that their itching gets worse at night and may sometimes get so bad that it makes them uneasy [10]. Malaise, anorexia and stomach discomfort are some constitutional signs of cholestasis that might appear. Steatorrhea may develop, and pale stools and black urine have both been documented [11]. ICP and other pregnancy-related illnesses such as acute fatty liver in pregnancy, hypertension during pregnancy/pre-eclampsia [12] and gestational diabetes [13] have occasionally been reported to co-exist. This highlights the variability of the disease's aetiology, and it is crucial to rule out other hepatic impairments in pregnant women from ICP. The biochemical abnormalities in obstetric cholestasis generally disappear within two to eight weeks after delivery, and it is not associated with ongoing liver impairment following pregnancy. In a few case studies, the course was longer, and the biochemical anomalies persisted for up to 34, 45, and 82 weeks after delivery [14]. It's crucial to rule out any underlying diseases in female patients with persistent liver impairment.

Foetal Findings

There is significant discussion in the works about the extent of the obstetric cholestasis-related foetal risk. The disorder has often been linked to poor foetal outcomes [15]. One series revealed a greater incidence of foetal problems in patients with hyperbilirubinemia than in patients with itching/pruritus alone [3]. Numerous research has aimed to link maternal blood serum levels with foetal outcomes. The sensitivity of bile acids as a predictor of prenatal risk has been investigated in various studies [16] involving a few patients [17]. Bile acids are frequently related to the origin of the foetal disorder. Long-term consequences of ICP are also linked to cardiovascular issues, cancer [18], hepatobiliary conditions [19] and autoimmune illnesses. Various outcomes are described ahead.

Meconium excretion in the amniotic fluid: Meconium staining of amniotic fluid (MSAF), which arises in around fifteen per cent of standard term deliveries, is thought to indicate foetal distress. Around 16%-58% of all obstetric cholestasis cases have been documented to have MSAF and up to 100% of cases have intrauterine death [20]. Pregnancies with increased maternal serum bile acids values had more prevalence of MSAF [21]. This is highly important because altering secretory phospholipase A2 can cause lung damage when the foetal lung is exposed to toxic levels of these molecules [22]. Animal models have shown that exposure to pathologically high amounts of bile acids has an adverse effect on normal foetal development, specifically on hepatobiliary function [23], with lasting problems for several weeks after delivery. The bile acids might also result in foetal distress and eventual meconium passage. This leads to meconium aspiration where the foetus consumes the meconium during birth. This blocks the airway and can cause severe respiratory problems.

Cardiotocography (CTG) abnormalities: Obstetric cholestasis has been linked to antepartum and intrapartum CTG abnormalities such as decreased foetal heart rate variability, bradycardia and tachycardia [24]. Recently, a case report has recorded foetal tachyarrhythmia causing atrial flutter during labour at thirty-seven weeks gestation [25]. Individual newborn rat cardiomyocytes exposed to taurocholic acid exhibit a reversible reduction in contraction rate. Taurocholic acid causes the cells to lose their capacity to beat rhythmically and exhibit aberrant calcium dynamics, which suggests that the CTG abnormalities seen may be caused by high bile acid levels in obstetric cholestasis [26].

Preterm labour: Although some researchers have found that spontaneous preterm labour occurs in up to 60% of births, the majority of the studies done at the same estimated rates of 30%-40% in obstetric cholestasis instances without active care [27]. Patients who have ICP frequently have premature labour and delivery. This happens because bile acids increase the uterus' sensitivity to oxytocin. Oxytocin is a hormone that stimulates uterine contractions. About 20 to 40 per cent of ICP pregnancies end in preterm labour spontaneously [10]. As bile acid levels rise, the danger also rises. While inducing early in most instances of ICP is seen as part of active therapy, premature delivery has some risks. Early contractions and cramping are indicators of premature labour, and the patient should call the doctor to be evaluated if these symptoms are present.

Respiratory distress after birth: Babies born to mothers with ICP at the same gestational age are nearly three times more likely to have respiratory problems at delivery than babies delivered to mothers without ICP [10]. According to recent research, high bile acid levels can interfere with the normal synthesis of a substance called surfactant, which aids in developing a baby's respiratory system after birth. This risk seemed to be enhanced significantly when ICP was discovered early and when bile acid levels were greater [28]. Delivery should be scheduled at a facility that can handle these infants because there is a greater probability that the baby may be admitted to a neonatal intensive care unit (NICU) after birth.

**Figure 1:**
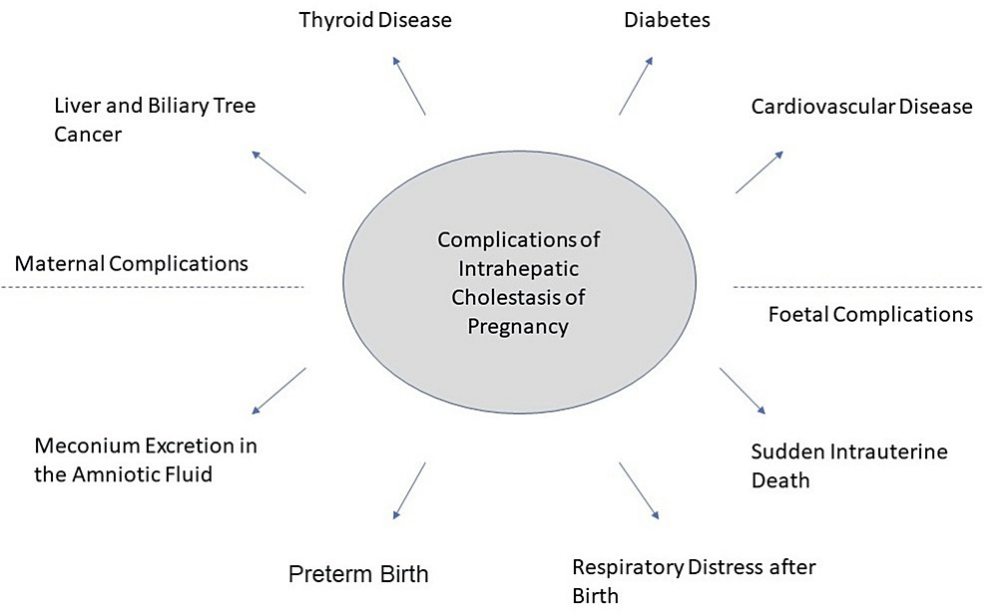
Complications of Intrahepatic Cholestasis of Pregnancy

Sudden intrauterine death (IUD): According to a recent study, there is a 1-2 % greater risk for ICP for bile acid above 40 mol/L, which is considered related to poor foetal outcomes [21]. Therefore, it is probable that ICP pregnancies with severe hypercholanemia have a more considerable IUD risk. Numerous earlier research has demonstrated that an ICP-complicated pregnancy has more chance of stillbirth. After thirty-seven weeks of pregnancy, the majority of these stillbirths take place [10]. Although the reason for the stillbirths is unknown, it is believed to be either a foetal cardiac rhythm disorder or a restriction of blood circulation in the placenta brought on by the high bile acid levels. Foetal monitoring cannot always predict the abrupt nature of stillbirth. The chance of stillbirth rises as bile acid levels rise, according to a recent study that examined more than 5000 cholestasis-related pregnancies [21]. These results provided comfort for patients with low bile acid levels since, as long as bile acid levels were below a hundred, the odds of stillbirth in cholestasis pregnancies were the same as in normal pregnancies. Bile acid levels beyond a hundred have a risk of 3.44%, bile acid levels between forty and ninety-nine have a chance of 0.28%, and bile acid levels below forty have a chance of stillbirth of 0.13% [16]. Figure 1 shows some maternal and foetal complications of ICP.

Investigations

Pregnancy-related abnormal liver function tests (LFTs) must be appropriately interpreted to prevent diagnostic errors. Both the mother's and the foetus' outcomes may be significantly affected by the underlying cause [29].

Liver Function Tests

In normal pregnancy: It is advised to apply modified upper limits in normal. For serum aspartate transaminase/serum glutamic-oxaloacetic transaminase (AST/SGOT) and serum alanine transaminase/serum glutamic-pyruvic transaminase (ALT/SGPT), the upper limit of the normal reference range is decreased by 20%, and γ-glutamyl transpeptidase (GGT) levels are decreased by a comparable amount in late pregnancy. Total and free bilirubin also decreases in all three trimesters, while in the second and third trimesters, a decrease in conjugated bilirubin is seen [30].

In obstetric cholestasis: Elevated serum levels of the transaminase enzymes are found in hepatocytes, hence a sign of hepatocellular injury. ALT/SGPT and AST/SGOT may increase in ICP either before or after serum bile acids rise [31]. Alanine transaminase (ALT) exhibits a 2-10-fold rise in serum values and is more significant than the rise in AST/SGOT [16]. Most ICP patients have normal bilirubin, which is of little use for diagnosis. It usually manifests as conjugated hyperbilirubinemia if increased [31]. Although GGT is usually normal [16], several investigations have revealed that it is elevated [32]. Although alkaline phosphatase (ALP) levels may increase in obstetric cholestasis, placental isoform is produced in huge quantities, making this biochemical marker of poor diagnostic relevance. Following acute hepatic injury, glutathione S-transferase alpha (GSTA), a phase II detoxifying enzyme, is quickly released into the bloodstream. It is a more accurate and precise indicator of hepatic dysfunction than conventional LFTs [33]. Table 1 illustrates the liver function test values in normal pregnancy and ICP.

**Table 1:** Comparison Between Liver Function Tests in Normal Pregnancy and ICP ALT- Alanine Transaminase; AST- Aspartate Transaminase; ALP- Alkaline Phosphatase; HDL- High-Density Lipoprotein; LDL- Low-Density Lipoprotein

Laboratory Tests	Normal Pregnancy	ICP
ALT	Normal	Increased
AST	Normal	Slightly increased
ALP	Normal	Increased
Total serum bile acids	Slightly increased	Increased
Cholic acid	Normal	Increased
Chenodeoxycholic acid	Slightly increased	Increased
Plasma glucose	Normal	Increased
Lipoprotein X	Not present	Present
HDL- cholesterol	Normal	Slightly decreased
LDL- cholesterol	Normal	Increased

Bile Acid Levels

The chief means of excreting cholesterol is through chenodeoxycholic acid (CDCA) and cholic acid (CA), which are the by-products of hepatic cholesterol metabolism. Some secondary bile acids are DCA (deoxycholic acid ) and LCA (lithocholic acid).

In normal pregnancy: As gestation progresses, total serum bile acids slightly rise in a healthy pregnancy. Individual bile acid levels have been examined in studies, and the results demonstrate that DCA remains unchanged, whereas CDCA increases by term. There are different reference ranges for total serum bile acids in pregnancy. Most authors agree that the upper limit of normal is between 10 and 14 μmol/L [10]. 

In obstetric cholestasis: With the CA levels [34] or the CA: CDCA ratio being suggested as the most sensitive indicators for the early detection of ICP [31], serum bile acid measurement is currently thought to be the most effective biochemical marker for the diagnosis and monitoring of ICP [35]. Although to a lesser extent, levels of DCA also increase, which indicates that the enterohepatic circulation is compromised. Some literature has instances of serum bile acid increasing to a hundred times the upper limit of normal value [36]. There is also disagreement about whether a spike in serum bile acids occurs before symptoms appear. There are cases where biochemical abnormalities or symptoms occur before the patient has high serum bile acids [31]. There is also a disagreement about whether serum bile acids should be tested post-meal or during fasting. It has been suggested that using a standard test meal may assist in identifying mild versions of the condition from normal since consumption of a standard test meal increases serum bile acids, notably CA, more dramatically and for a more extended period in ICP patients than in control women [37]. However, if utilized in regular obstetric practice, this strategy would be time-consuming and expensive.

Other Tests

Lipids: Deranged lipid profiles have been linked to ICP in cross-sectional investigations [38], and higher levels of total cholesterol and apolipoprotein B-100 were discovered in future longitudinal research [39]. 

Glucose: According to a study, obstetric cholestasis is linked with impaired glucose tolerance. In ICP, the oral glucose tolerance and two-hour post-prandial glucose tests result were more [40].

Urine

Analyses of the urine from ICP-afflicted women reveal lesser elimination of DCA and LCA but higher elimination of total bile acids, with CA and CDCA excretion increasing 10- to 100-fold. Reduced secondary bile acid excretion is consistent with decreased enterohepatic circulation and lends credibility to the notion that a canalicular abnormality is a crucial component of ICP. ICP with anomalies in the serum can be detected or ruled out by urinary bile acids. Although co-detection of progestin sulphates is reported, urine bile acid sulphates show high specificity than non-sulphated urine bile acids at equal sensitivities [41].

Liver/Gallbladder Ultrasound Tests

Thirteen percent of ICP-afflicted women report having gallstones [42]. There haven't been any studies comparing ICP patients to controls, and pregnancy is linked to an increased prevalence of asymptomatic cholelithiasis, even though have a higher vulnerability to cholelithiasis [43]. Cholelithiasis rates are also more significant among the direct blood relatives of distressed women.

Management/treatment

The objectives of management are to lessen the mother's symptoms and biochemical deviations and the risks of foetal distress, premature birth, and abrupt foetal death. Once the diagnosis is confirmed, the patient should be continuously watched by the obstetrician and nurse to make sure the ICP is not becoming worse. In order to effectively treat ICP, an interprofessional team approach is required. A typical interprofessional team should, where possible, include an obstetrician, a specialist in maternal-foetal medicine, a gastroenterologist, an anaesthetist, and the nursing staff.

Foetal Monitoring

Numerous case reports describe normal foetal movements or CTG in the hours before foetal demise [44]. Therefore, it is widely believed that current foetal surveillance methods do not prevent IUDs. When they are carried out, the doctors should console the women suffering from ICP. When recognizing the unique risk of foetal impairment in pregnancies affected by intrahepatic cholestasis, a Doppler examination of the umbilical artery may be helpful. According to one research, routine amnioscopy at thirty-six weeks, together with routine monitoring for the well-being of the foetus, led to favourable foetal and neonatal results [45]. However, many obstetricians could view this strategy as being excessively intrusive.

Antepartum Monitoring of ICP

Routine antepartum foetal testing has not been shown to be effective in individuals with ICP in identifying foetuses at risk of miscarriage. There is currently no evidence that any prenatal examination can predict or lower unfavourable perinatal outcomes. However, the majority of healthcare professionals found it acceptable to perform routine prenatal testing on individuals with ICP. The general opinion is to employ weekly biophysical profiles (BPP) to identify foetal impairment, despite the lack of high-quality data supporting prenatal screening in patients with ICP [46].

Elective Delivery/Timing of Delivery

For patients with ICP, several experts have recommended elective early delivery to lower the chance of unexpected foetal death. The danger of foetal mortality against any potential hazards associated with preterm should decide the timing of delivery [46]. Delivery should most likely take place by 35 to 36 weeks of gestation in women with total bile acid values of 100 micromol/L or above [47]. Women with ICP whose jaundice and maternal symptoms are not alleviated by medicine require increasing dosages of UDCA, and those with prior history of IUD due to ICP before 37 weeks or more than 100 micromol/L of total bile acid concentration in serum, should consider delivery earlier than 37 weeks of pregnancy [48]. Studies have found positive results when labour is induced at 37 or 38 weeks gestation [45]. Due to the IUDs' tendency to cluster at later stages of pregnancy, several practitioners in the UK have accepted this technique. However, there haven't been many reports on the week of gestation at which the IUD develops, and there haven't been any sizable potential studies about whether pharmacological therapy or early delivery can avoid unfavourable foetal outcomes.

In ICP patients, caesarean delivery can be done. In the first two to three days after birth, pruritus usually disappears, and serum bile acid levels gradually return to normal levels. Breastfeeding is not prohibited in ICP, hence women with ICP are still permitted to breastfeed. To assure resolution, postpartum bile acid monitoring and liver function assessments should be performed in 4-6 weeks. Women who continue to have abnormal LFTs beyond 6 to 8 weeks need to be looked into for other causes.

Complications

One of the dangerous risk factors for the abrupt death of the foetus is ICP, which poses a serious risk to the foetus. Intrahepatic cholestasis of pregnancy is also linked to a higher risk of poor obstetric outcomes [46]. High body mass index and dyslipidemia at the age of 16 were shown to be more likely in children born to women with ICP [49]. In succeeding pregnancies, ICP has a significant recurrence rate. Through the placenta, maternal bile acids are transferred to the foetus and deposited in the amniotic fluid to cause abnormalities. Preterm labour is more common in women with ICP; the explanation is unknown, although it might be because of bile acid deposition in the uterine myometrium, which leads to an increase in uterine activity. The most concerning side effect of ICP is sudden IUD. Although the exact cause of foetal death is not known, it may be related to the toxic effects of bile acids on the developing foetus's heart, which can lead to arrhythmias, and chorionic vasospasm, which prevents oxygen-rich maternal blood from reaching the developing foetus, which can result in asphyxia [50].

Drugs

Ursodeoxycholic acid (UDCA): The biological bile acid pool in humans contains around 3% of the naturally occurring hydrophilic bile acid known as UDCA. It is used in the treatment and ICP has been utilized successfully for many years to treat primary biliary cholangitis and other cholestatic illnesses. There is proof which shows UDCA increases biliary secretion by regulating the bile salt export pump (BSEP) and the alternative exporters multidrug resistance-associated protein 3 and multidrug resistance-associated protein 4 post-transcriptionally [51]. Additionally, it lessens cytochrome c expression and mitochondrial membrane permeability to ions and it has antiapoptotic properties [52]. The serum concentration of ethinyloestradiol 17-glucuronide, a significant cholestatic metabolite of oestrogen, is also decreased by UDCA. Additional case studies published prove that UDCA therapy improves the clinical and biochemical state of ICP [53]. Rifampin, cholestyramine and S-adenosyl-L-methionine (SAMe) are examples of other drugs that can be used for individuals whose ICP has not improved and who are resistant to UDCA.

Dexamethasone: By decreasing the foetal adrenal glands' production of the precursor, dehydroepiandrosterone sulphate, dexamethasone prevents the production of placental oestrogen [54]. Dexamethasone has been employed often to enhance foetal lung maturation and is said to be safe in this scenario. It can, however, easily penetrate the placenta, and there is evidence from both human and animal studies suggesting repeated high dosages are linked to aberrant neural development [55] and smaller babies with decreased weight [56].

Rifampicin: Rifampicin has been used successfully in treating various conditions of the liver, like gallstones, primary biliary cholangitis [57] and primary biliary cirrhosis, despite the fact that there are no published trials documenting its usage in ICP [58]. In these investigations, rifampicin therapy led to a substantial drop in blood levels of total bile acids (TBAs) and transaminases as well as provided relief in pruritus, indicating that it may also be effective in treating ICP. Rifampicin has been demonstrated to improve bile acid detoxification, which is complementary to the up-regulation of bile acid export brought on by UDCA [59]. This finding raises the possibility that using the two medications together may be more beneficial than using either one alone.

Vitamin K: A decline is present in the uptake of bile acids at the terminal part of the ileum because of their decreased enterohepatic circulation, which causes a decrease in the absorption of fat-soluble vitamins linked to ICP. Because of this, many doctors decided to give women oral vitamin K to prevent any potential risk for foetal antepartum and maternal intra- or postpartum haemorrhage [60]. But no research has yet been done so there is no confirmation or disapproval of the technique.

Others

Early trials of SAMe for the treatment of ICP suggested that it improved the symptoms; this information was later corroborated in placebo-controlled research including 15 women who received high dosage SAMe. SAMe appears to be well tolerated, and no further negative maternal or foetal consequences have been noted except for issues with peripheral veins, following extended intravenous treatment [61]. SAMe is a less desirable alternative for the treatment of ICP since it is frequently delivered using a twice-daily intravenous regimen. In one trial, UDCA was shown to be more effective than SAMe in terms of improving liver function tests while being similarly effective at improving pruritus [62]. Better results are seen when SAMe is used with UDCA but the effect of this combination on the foetus is still not clear [63]. An anion exchange resin called cholestyramine works by binding bile acids in the stomach, slowing enterohepatic circulation, and boosting bile acid excretion through the faeces. According to various studies, cholestyramine is useful for lowering pruritus in ICP [64]. Aqueous cream with 2% menthol used topically is useful for relieving itching, but it has little effect on underlying biochemical problems.

## Conclusions

This is a review of the foetal consequences of ICP and the treatment strategies for its avoidance. ICP is associated with maternal distress and foetal complications. Although ICP is generally limited to the mother, it is known that ICP-affected pregnancies have a higher risk of foetal problems. There are also higher chances of foetal discomfort, meconium-stained amniotic fluid, preterm birth, and IUD. A higher risk of prenatal problems may exist in people with hepatic or biliary disorders. Diagnosis is mainly done by bile acid estimation and LFTs in the mother. Bile acids and liver enzymes are significantly increased in ICP. As the levels of maternal bile acid increase, the risk of foetal complications also increases. Currently, there are no tests that can diagnose foetal complications. Foetal development is assessed through ultrasonography and CTG findings. Patients with ICP and increased TBA levels should have a BPP every week for foetal health monitoring. Delivery between thirty-six and thirty-seven weeks should be taken into consideration to prevent foetal problems in ICP patients. When ICP is diagnosed, UDCA is used as part of the current drug therapy. For treating maternal symptoms, ursodeoxycholic acid (UDCA) has been proven to be more effective than other treatment techniques, such as the use of SAMe, cholestyramine, and dexamethasone, and is not harmful to the foetus. Symptoms of pruritus usually disappear after pregnancy. Infants born prematurely are generally admitted to the NICU because of preterm labour. They may have more health problems but can recover in the NICU.
